# Atlanta Society of Medicine

**Published:** 1889-03

**Authors:** 


### Society Reports.

### ATLANTA SOCIETY OF MEDICINE.

MEETING JANUARY 15, 1889,
PRESIDENT HUNTER P. COOPER, M. D., IN THE CHAIR.

Dr. W. P. Nicolson read notes on "Ununited Fracture of the Fore-arm." He said that he desired to have it understood that he would not read a paper on "Ununited Fractures," as the notice of the Secretary would lead the members to suppose, he only wished to read some notes on a case of ununited fracture of the fore-arm. The Doctor then read from his notes that Phil Sutton, white, male, aged 42 years, in March 1888, fell from the platform of a horse-car and sustained a fracture of the left fore-arm, caused by the wheels of the car passing over it. The fracture was dressed by a physician in the neighborhood. On the following night he took charge of the case, at the request of the Street Railroad Company. Upon examination, found the fore-arm bandaged to anterior, and posterior board splints extending from ends of fingers to elbow; a high degree of inflammation and swelling of the soft parts; vesicles and bullæ on the hand and the arm. Made no change, simply cut the bandage which was binding the swollen hand. The following day, on removal of the bandage and splints, he found the entire fore-arm very much swollen and covered with vesicles. Learned from the patient that the physician who had dressed the injury, had pronounced both bones fractured just above the wrist; said, as far as he could judge, the fracture had been reduced and the bones well co-aptated. Owing to the condition of the arm he did not think further examination or manipulation necessary or justifiable. After dressing with antiseptic gauze, he bandaged the arm to an anterior splint, and directed the bandage to be kept wet with an antiseptic lotion for a few days. Shortly after this he removed the straight anterior splint, and placed the arm upon an angular splint, extending from the axilla to the fingers. Everything seeming favorable, about the fifth week he instituted passive rotary movements, and continued it at intervals some two or three weeks, with the result of re-establishing movements of pronation and supination. The patient was then dismissed, with instructions to call at office once a week. A short time after being released from the bandages, the patient called his attention to motion at seat of fracture, that could be elicited by pressing the hand upon a table and contracting the muscles of the fore-arm. This movement, doubted at first, was so marked at his next visit, that he again placed the arm upon the right-angle splint. Three weeks confinement upon the splint, with attempts at friction between the fragments, resulted in no improvement. He then applied a plaster Paris bandage, which was worn for some weeks; during the time, the patient attempting to work and getting on a spree—the result of the treatment was negative. After this, the patient, constructing a leather splint which he could lace over the fore-arm, did some work, but did not resume his occupation. Becoming anxious about the usefulness of his arm, the patient, he said, again called upon him and insisted upon having him perform an operation to restore, if possible, the union of the bones. The doctor then detailed the operation as follows:

"November 24th, 1888, in the ampitheatre of the Southern Medical College, in the presence of the class, and assisted by Drs. Hagan, Divine, and others, the operation was made. Upon examination there was found a point about two inches above the wrist joint, almost as movable as that joint, and extending through both bones. There was some decided thickening of the soft parts, causing a projection over the back of the radius. It was determined to cut down upon the false union, remove the intervening tissue and wire the ends of the bones. An incision four inches long was made over the anterior border of the radius down to the bone. There was some deficiency in length of the incision at the lower part, on account of fear of wounding the radial nerve. The upper end of the lower fragment projected prominently about two inches above the wrist joint, and from this an oblique line of fracture passed downwards and outwards towards the ulna. There was complete fibrous union, binding the fragments closely and firmly together, so that their relative ends could not be displaced, though enough to permit free flexion. A strong cartilage knife was thrust between the fragments, and by pressing them apart, the fibrous tissue was cut through with the point of the knife. Then with chisel and rongeur, this was forced from the bone until crepitus could be elicited by rubbing the fragments with the parts held in position. A Brainard drill was passed first through the projecting point of the lower fragment, and then obliquely through the edge of the upper fragment, and through the opening a silver wire was introduced and loosely twisted. The suture was not passed very deeply into the bone on account of being unable to very widely displace the fragments. An incision was now made over the inner-border of the ulna, corresponding with that in the radius. The patient was now taking ether so badly that I concluded not to wire the ulna fragments, but to trust to their being held in apposition by the splint. The radial wire was now tightened and twisted, and the tourniquet removed. There was only capillary oozing, so the wounds were immediately closed with cat-gut, and drainage tubes inserted. During the entire operation antiseptic details were strictly adhered to, except in one instance which will be mentioned later. The entire fore-arm was now enveloped in a gauze dressing, and over all a plaster of Paris bandage was applied, extending from the tips of the fingers to the upper part of the arm. On the following day the temperature reached 102 degrees, there was considerable pain at the seat of operation, and some staining of the plaster bandage by blood. The temperature gradually declined until it reached 99 degrees; but little pain after first three days. On the 12th day following the operation, there was a sharp rise of temperature to 102 degrees, and the patient informed me that some pus was oozing through the bandage. Assisted by Dr. Hagan I removed the bandage, finding the dressing about the radial wound saturated with pus, and the wound, on account of yielding of sutures, gaping open. The ulna wound was free from suppuration; upon the back of the forearm, between the bones, was a fluctuating swelling, evidently pus, but not connected with either wound. Thinking this would find its way of exit through one of the wounds, it was not opened. The drainage tubes were removed and a gauze dressing, with plaster of Paris over all, re-applied. In spite of the disagreeable result in the radial wound, there was evident bony consolidation of marked character. Six days after, and on account of great pain, the dressings were again removed, when the pus accumulation on the posterior surface were larger and the skin over it red and tense. The ulna wound was healed perfectly dry, and that over the radius was filled with healthy granulation. The abscess on the back was opened and drain inserted, and the fore-arm in a gauze dressing was placed on an anterior board splint. There was increased strength of bony union. This splint was worn about ten days, when upon removal of dressings, the wounds were found almost healed, and the pus cavity so small that the tube was removed, but the bony union appeared to have yielded some. Two days later, December 25th, in my absence from the city, an excellent plaster of Paris bandage was applied for me by Dr. Hagan. This was worn until January 12th, (1889), when it was removed, and the wounds found all soundly healed and bony consolidation practically perfect. As a matter of precaution a Levis bone splint was applied and worn till to-night." Dr. Nicolson then introduced the patient, removed the bandages and splint. The arm presented a very excellent appearance and was examined by Drs. Westmoreland and Cooper, who could detect no movement whatsoever. Dr. Cooper asked if the patient had the motions of pronation and supination.

Dr. Nicolson replied that, as yet, he had not allowed him to practice either; preferred to have him wait awhile. Dr. Nicolson further said, that, as regards the suppuration, that it was unexpected, and that he could only account for it in this way: that one of the gentlemen present at the operation, did not disinfect his hands when requested to examine the bone, after denudation had been accomplished. That the question was, what was the cause of failure to secure bony union? Cases occur in which the same means were used and no trouble. Other cases, where bones were not at all in apposition, became united. In re-sections, many times, even when passive motion is practiced, bony union will occur. He said that: "The principal factors in preventing bony union, were divided into local and constitutional. Among the former, we have malposition; motion, due to insufficiency of confining apparatus; injury to the nutrient artery of the bone; the interposition between the fragments of pieces of muscle, tendon, etc. Among the constitutional we have: Pregnancy, syphilis, exhausting disease and intemperance. The failure to unite may be of different character. Bony material may not be thrown out at all, resulting in the absorption and rounding of the ends of the bone, and the formation of a "false joint." Again, the bony callus may be deposited, and, from some cause, re-absorbed, leaving only a fibrous connection. Lastly, the lymph thrown out may not reach the bony stage at all, but remain fibrous. Among the causes leading to the absorption of bony callus there have been mentioned many exhausting diseases, especially typhoid fever; but usually its results prove too early use of the limb, especially the lower extremity, when there is much pressure. I am inclined to believe that the cause in the case I have related, was the too early use of the limb—though, usually, not so much time as was given in the case is necessary. Probably the life-long intemperance of the man (for he had been a drinking man from his youth up to the present time) entered as a large predisposing factor."

The discussion was opened by Dr. Willis Westmoreland, Jr., who said that he had listened with pleasure to Dr. Nicolson's excellent paper. The subject was a very important and peculiar one. A man should always withold judgment, for some cases occur wherein good results are expected and are not obtained, and in others, when trouble is apprehended, the case passes on to a favorable termination. He mentioned a case in point, of a patient who had his leg fractured by a car wheel running over it, and who had had syphilis; said, in this case he expected to have failure of union of the fracture but did not, the bone united and the man has a good leg. He thought that there was no such condition as absolute non-union. He would divide the subject under discussion into two classes, viz: those in which the union was arrested at the fibrous stage, and those in which cartilage had been formed. He thought the case reported by Dr. Nicolson belonged to the latter class. The causes of the arrest of union were sometimes very obscure; syphilis and defective innervation were sometimes very prominent causes; that keeping the patient quiet and in bed sometimes produced a phosphatic deposit in the urine, which indicated that a condition existed which was retarding the repairing process; this could be overcome by quinine, mineral acid, good diets and getting the patient up, and insisting upon gentle and moderate exercise, when possible.

His plan of treating fracture was to immobilize the fragments, as soon as possible with a plaster of Paris bandage. He did not, in fracture of the leg, go above the knee, or below the ankel with the bandage; ordered his patient to walk about as soon as the bandage was firm. This moving around furnished the necessary irritation to bring about good results. He never had had a failure of union in any of his cases.

Dr. Westmoreland mentioned the case of an engineer, who had a fracture of the leg, in which the process of repair was seemingly arrested at the fibrous stage; he treated it with a plaster of Paris bandage and directed the patient to resume his occupation, which he did with no inconvenience, and his limb was soon restored to usefulness.

Another case of a farmer with a fracture of both bones of the leg, in which there appeared to be a cartilaginous formation and free motion of the fragments; instituted the same treatment, and had a perfect union after the application of about five or six plaster Paris bandages, during a period of some six months.

He did not think this treatment as applicable to the arm, as to the leg or foot, because the same character and degree of motion could not be had.

He then exhibited Gowan's instrument, modified by Wyeth, for re-section of the ends of fractured bone; said its advantages were, that it required but a small external incision, and that the ends of the bone could be cut off without having to pull them through the incision; could be used on both sides; he had re-sected a rib with it and found its action very satisfactory; that with regard to operation for ununited fracture, he liked using Wyeth's instrument and leaving the drill in position; next, the wiring of the ends of the bones, as to seton, etc., did not like anything of the kind.

Dr. F. W. McRae said that he thought general medicine ought to be brought into use in the treatment of these kinds of fractures under discussion. He mentioned authors who allude to the phosphatic deposit in the urine—as spoken of by Dr. Westmoreland in this connection—as showing a condition of the system that required aid; suggested the use of phosphorus as a good and powerful adjuvant in these cases, especially when occurring in children.

Dr. Duncan thought that the too free use of cold applications, and the too tightly bound splints, were frequent causes of non-union.

Dr. F. O. Stockton remarked, that he thought the dental engine would be of great assistance in drilling the holes through the ends of the bones, and do it very accurately. He said he had seen two surgeons' engines for this purpose. An ingenious one was that invented by Dr. Bonwill, who also had a splint which he used in these fractures. His splint consisted of a steel bar, (the Dr. did not remember the dimensions,) with holes at either end. Through these holes were passed steel rods. The bars had thumb-screws on either end; an incision was made over seat of fracture, and holes drilled through the ends of the bone, with the engine drill. Then this steel bar was placed over the incision, and the steel rods passed through bar and bone. On each end of the rods were buttons which were screwed up, and which held the rods firmly in the bone and bar. The thumb-screws would then be tightened, and thus bring the ends of the bones together, and hold them securely. He thought this a very advanced method of treating ununited fractures.

Dr. Cooper said he had had a case of ununited fracture of the tibia and fibula, in a little negro girl, aged three years. He learned from the mother that the midwife had used force in extricating the child, which was being born feet foremost. The mother noticed nothing wrong with the child until she attempted to walk, then she saw something was the matter with one of her legs. Dr. Cooper said as soon as he examined the child, he readily detected fracture at the junction of the middle and lower third of the leg, and that the foot was inclined at an angle of 45°. He operated, with the assistance of Drs. Nicolson and Greene, taking antiseptic precautions; made an incision over and down to seat of fracture. Found the bones imbedded in dense fibrous tissue; cut this all away, and divided the tendo-achillis; did not wire the ends of the bones. Used antiseptic dressing and plaster of Paris bandage, but failed to get any result. Tried making the child walk, but with no benefit. He said the child was now wearing a steel splint, with which she could walk very well. Dr. Cooper laid stress upon the fact that physicians and surgeons could not be too guarded in criticising their fellow-practitioners; that mishaps are liable to happen to each and all, when there is no accounting for them. He instanced a case of a New York surgeon, who had a patient twenty-five years old, hale and healthy, with fracture of arm that did not unite, nor could be made to do so. He said he could not see, nor could any one tell, why such a failure should occur in a healthy man. He added, in conclusion, that he thought that sometimes too free use of antiseptics had a great deal to do in weakening the repairing power of the parts.

Dr. Nicolson, in closing the discussion, said that he was glad the subject had been so thoroughly discussed; he had thought of using the dental engine in this case, but did not get one. Again, he was very anxious to use the drill with the aid of cocaine, and leave it in position, but that the patient insisted upon his "cutting his arm." He had thought when he saw the pus, that the wire in the bone would give trouble, but in this he was agreeably disappointed. Said he could not tell if having the pus did any harm, but that it was very disagreeable and unexpected; that this case taught a lesson, that physicians and surgeons should be very careful how they blame each other, that such unlooked for results may possibly happen to any one. When he first saw the arm it was a bad one, the fracture had been properly reduced, that his impression is, as he had said before, that the non-union resulted from the patient using his arm too early. He remembered a case of fractured arm which had united by bony union, when suddenly as it were, the bony portion entirely disappeared leaving only a fibrous connection. No cause could be assigned; the man was healthy and the conditions favorable, and yet the entire bony portion of union was absorbed. So in many cases, as in this, the cause of failure of union could not be found or explained. He expected his patient to have a strong and useful arm; and concluded by thanking the members for coming out so strongly on such an inclement evening, he appreciated the compliment paid him by their presence.

---

MEETING, February 5th, 1889.

Dr. Hunter P. Cooper, President, in the chair.

DERMATITIS OF BOTH EYE-LIDS, BROUGHT ON BY THE APPLICATION OF TINCTURE OF ARNICA.

Dr. J. M. Crawford reported a case of a man of fifty who while drinking, fell and injured his face and eye-lids, breaking the skin. Was told by a friend to use pure tincture of arnica. He saturated a cloth with the tincture of arnica undiluted, and applied it to his eyes, kept it on for a half-hour. Two days after Dr. Crawford was sent for, and found the lids very much thickened, so much so that he could not raise them, and consequently could not get a view of the eye-balls. Dr. Crawford thought the eye-lids to be about one inch in thickness. Two days subsequently, sloughing of the whole integument from eye-brows to tarsal cartilages of both lids took place, and in one eye there was extensive effusion and detachment of the retina. The other eye was not so affected, and was eventually restored to usefulness. Dr. Crawford said that he had never seen or heard of a case of the kind before, but that he knew that arnica was very irritating in full strength, and very dangerous when used about the eyes.

HÆMATOMA OF THE THIGH, RESULTING FROM A PISTOL WOUND.

Dr. Gaston reported a case of a man of fifty who had received a pistol wound of the right thigh, some nine weeks previous to his seeing him. On examination of the limb, found the ball had entered at junction of lower and middle third, ranging obliquely upwards across the inner portion, and had been cut out shortly after the accident. A large tumor occupied track of the ball, indicating that there had been great infiltration of blood in the tissues surrounding track of wound. Septic contamination had seemingly already begun. Dr. Gaston, determining to operate, applied tourniquet and laid open the tumor, making an incision of eight inches in length, upwards from point of entrance of the ball, and scraped out with his hands an immense accumulation of clotted blood, the femoral artery was found to be open to the extent of half an inch, blood escaping freely. A strong iron dyed silk ligature No. 13, was tied three-quarters of an inch above and another at similar distance below the opening. The dense condition of the femoral walls prevented these from entirely closing the lumen of the artery and two other silk ligatures were applied, one immediately above and below the opening, which controlled the bleeding.

The cavity was thoroughly washed out with a warm solution of chloride of sodium, followed by a carbolic solution, antiseptic gauze saturated with turpentine f ℥i, and camphor f ʒi, was placed in the cavity, and two drainage tubes inserted, so as to drain from upper and lower extremity of the incision. A single stitch was put in near each tube to retain them; the intervening portion left open to admit free exudation from deep seated parts. Over all, iodoform and antiseptic gauze, covered with absorbent cotton, secured by a roller bandage. The lower extremity entirely enveloped with cotton and supported by a roller; hot bricks to leg and foot ordered.

November 22, dressings were removed, and the cavity again washed out with a solution of chld. sodii, followed by carbolized water, and an antiseptic gauze, covered with iodoform, placed in line of incision; the thigh again covered with cotton and bandaged; no diminution of the temperature of limb; general œdema verymuch lessened.

November 29, the general condition of the patient favorable. One tube removed; injection of peroxide of hydrogen used to correct purulent collection.

December 12, suppuration notably diminished under influence of peroxide of hydrogen; more healthy; ligatures had not separated.

Dr. Gaston passed an aneurism needle within the loops of the two upper ligatures and cut them away, to the two lower ones, which were covered by adhesion of the skin, he attached a piece of rubber tubing, secured to the thigh by an adhesive plaster, to keep up constant traction, and put the patient on mur. tinct. of iron, and tinct. nux vomica.

December 19, patient was improving; ligatures still remaining.

December 22, ten days' traction not bringing away the ligatures, Dr. Gaston used an ordinary button-hook for securing the loops, and cut them away with a narrow bistoury, each loop measured one inch and a half. The patient continued to improve and now requires no surgical attention.

Dr. Gaston further remarked, that he had treated other cases of this kind with similar success, that the favorable results certainly encouraged the evacuation of the collection of blood from injuries of the femoral and other arteries of the extremities, and the ligation of the arteries so injured, for the prevention of septicæmia, even after a lapse of some time from the infliction of the injury.

Dr. Gaston concluded by saying, that he thought that the man (whose history he had just related) had escaped death by hemorrhage, from the fact that his limb was bent at the time he received the shot, and on straightening it out the muscle acted as a valve to the external wound, thus preventing any excessive external bleeding.

### REPEATED ABORTION.

Dr. Noble reported a case of a patient who had presented herself to him, with a history of three successive abortions; was at the time of her visit four and a half months advanced. On examination, he found the uterus acutely anteflexed and jammed in the pelvis; body contracted; two posterior ligaments very tense and somewhat enlarged; there was considerable venous stasis; mucous membrane black; pouting of the lips of uterus, also much oozing from cervix; could not lift the uterus up, was afraid to make too great an effort on account of pain produced. Commenced treatment with small tampon of cotton and glycerine applied every twenty-four hours, and gradually increased in size until all engorgement was relieved, and the uterus could be lifted up and held in position without occasioning trouble. The patient returned home and has been progressing favorably.

Dr. Noble said that he had treated other cases, where there was extensive laceration of the cervix, and where the lips of the cervix were much swollen and rolling, with the same treatment, and had always had benefit therefrom; and that if this treatment was carefully and judiciously applied and kept up, that it would meet the indications in every case where there was no constitutional disease, or disease of the placenta. Said he had used this treatment where there was decided contraction of posterior ligaments, fixation and anteversion of the uterus, and free hæmorrhage, and had good results. His plan was to apply these cotton and glycerine tampons every twenty-four hours, until he was able to correct the position of the uterus. He did not pack the tampons tightly; only did so when the uterus was greatly prolapsed, and would stand the pressure of tampons so applied. He added, that he always interdicted coitus, and did not give any medicine unless the patient was run down; then he gave a tonic, or if he noticed any indication of syphilis, he treated them for that disease.

Dr. Bizzell thought Dr. Noble's treatment for such cases very excellent, especially the interdiction of sexual intercourse. Said that he always sent his patients away from their husbands, and thus, relieving them of a constant source of irritation, brought them out all right.

### DIPHTHERIA COMPLICATING SCARLATINA.

Dr. Duncan reported a case of a child, female—aged five years; When seen by him, November 6th, had very frequent pulse. temperature 104°; general diffused eruption; exudation over pharynx, uvula and nasal passages, completely blocking up the nostrils; larynx also involved; both eyes inflamed; diphtheritic exudation on side of left eye. November 10th, globe of left eye became affected; color grayish. Staphyloma supervened, and the eye-ball ruptured; sight lost; child also complained of earache; was delirious, and her respiration difficult. After a few days the fever declined; membranes detached, both from throat and nose; no tendency to reform; child thought to be doing well.

About 15th day a relapse occurred—temperature 102°—with recurrence of rash, the lids of the left eye took on inflammation, and were very much swollen. Abscess formed in lower lid, which was opened, and continued to discharge pus for some two weeks. Pus was discharged from both ears during third or fourth week. Desquamation began about third week; during second week albumen appeared in the urine; continued in quantity for a few days, then disappeared. The treatment adopted was of a tonic and stimulating character. Washes of bi-chloride and other antiseptics were frequently and thoroughly used. Dr. Duncan said he had great confidence in the iodine, iron and potash treatment, and he believed the bi-chloride did great service in this case.

### OBSTRUCTION OF BOWELS.

Dr. Bizzell reported case of a woman, aged 60. Sausage-shaped mass in region of ileo-cæcal valve, stercoraceous vomiting, collapsed. Introduced rectal tube two and a half feet; used one gallon warm salt water, with one ounce turpentine; gave brandy, rubbed abdomen with ointment of belladonna, camphor and mercurial ointment, and applied poultices to abdomen. Patient recovered. Dr. Bizzell said this patient had had repeated attacks of bilious colic; that it was his experience that habitual bilious colic very generally produced volvulus.

### EMMETT'S BUTTON-HOLE OPERATON FOR CARUNCLE OF MEATUS URINARIUS.

Dr. Hardon reported case of a woman aged 60, who suffered with symptoms of vesical irritation for two years. Urine healthy; no signs of cystitis. On examination of vulva, found a caruncle at meatus, which appeared to obstruct flow of urine. Patient said it had been removed some time before, but came back. Found also the old cicatrix. Determined not to remove growth, on account of its possible recurrence, but operated after Emmett, making a button-hole in the urethra three-quarters of of inch in length, and stitched urethral mucous membrane to mucous membrane of vagina. Used 15 per cent. solution of cocaine, locally to point selected, which acted so well, that the patient experienced no pain during the operation, no hemorrhage at time of making the slit. Second night after, some considerable bleeding from the upper edges of slit; controlled easily by a serrefine, the edges soon healed, and the patient has been greatly benefitted; can now retain her urine without inconvenience. Dr. Hardon said he thought that this operation of Emmett's was an excellent one. He had performed it several times, and would continue to do so, notwithstanding the fact that some of the Northern gynecologists were not in favor of it.

SCHEDER'S METHOD, AS APPLIED TO TREATMENT OF FRACTURED, AND NECROSED HUMERUS.

Dr. Westmoreland, Jr., reported case of boy, age 15, who had about one year ago, injured his shoulder while wrestling. The shoulder, at time of accident, was examined by local physician, and pronounced a dislocation. Dr. Westmoreland said that when the boy was brought to him, he found an abscess of the whole shoulder, extending to the arm-pit—he also found that the humerus had been fractured near the upper end. He let out the pus, put in drainage tube, and applied a splint to the arm—the boy then went home, but returned to him, two weeks after—he then concluded to treat the case by Scheder's method—put on an esmarch bandage, using antiseptics thoroughly and freely—cut down upon the bone and discovered three sinuses in the bone—an upper one, running up towards the shoulder-joint, the middle one running directly into canal of the bone, and a lower one running in same direction as middle sinus, but was the largest of the three—all were round—that this portion of the bone was denuded—between the upper and middle sinus he found two pieces of sequestrum. He removed all sequestra, and with the chisel and spoon, he cut and scooped away all the dead down to the sound bone, and also scraped off the pyogenic membrane, put in catgut sutures in the lower and middle portion of the incision, leaving an opening in the upper part, took off the esmarch, and allowed the wound to fill with blood. He then put on a rubber cloth, to prevent the dressing from absorbing too much blood; over this he placed a heavy dressing of antiseptic gauze, and bandaged all to the arm—this dressing he kept on, until such time as he thought the exuded blood had organized—took off the dressing, found the wound granulating, and everything progressing favorably.

Dr. Westmoreland then introduced the patient. The boy presented a good arm; the line of incision healed, there remaining only a slight fistulous opening, which Dr. Westmoreland said resulted from his neglect to remove sufficient tissue from around the original fistulous opening. He thought the boy would have a useful arm, and that method of treatment would be always successful, provided the strictest antiseptic precautions were taken.

Dr. Cooper introduced a patient with locomotor ataxia, simply to show the degree of ataxia—patient could not stand with his eyes shut—knee-jerk absent—could not control limbs when told to place them in certain position—had a shuffling, shaky gait. His eye-sight, however, was good, nor was there any impairment of his sexual functions.

Dr. Avary mentioned, that as coroner, he had been present at the autopsy of a man who, in life, had given no indication of cerebral disease. On examination of the brain a large cavity was found in the brain substance, near left lateral ventricle—on the left side, an old blood clot; on the right side, a gummy condition, and a collection of a substance similar to frog spawn, there were also signs of pachymeningitis ; cavity was empty, blood vessel seemed to have ruptured external to cavity.

Dr. Avary said he would like to hear remarks upon the probable cause of this condition, but the hour being late, the discussion was deferred to a subsequent meeting.

